# Children’s social emotional competence in Pakistan and Sweden: Factor structure and measurement invariance of the Social Competence Scale (teacher edition)

**DOI:** 10.3389/fpsyg.2022.1020963

**Published:** 2023-01-16

**Authors:** Sarah Thomas, Anna Kågström, Kyle Eichas, Ayesha Inam, Laura Ferrer-Wreder, Lilianne Eninger

**Affiliations:** ^1^Department of Psychology, Stockholm University, Stockholm, Sweden; ^2^Department of Public Mental Health, National Institute of Mental Health, Klecany, Czechia; ^3^Department of Psychological Sciences, Tarleton State University, Stephenville, TX, United States; ^4^Department of Humanities, COMSATS University, Islamabad, Pakistan

**Keywords:** factor structure, child development, Pakistan, Sweden, social emotional competence, Social Competence Scale

## Abstract

**Introduction:**

Social emotional competence is fundamental to the positive development of children and youth. Accurately understanding and assessing children’s social emotional competencies, using psychometrically sound instruments, are essential to global efforts to support children’s social emotional learning, academic achievements, and health. This study examined the psychometric properties of a teacher-reported measure of young children’s social emotional competence, the Social Competence Scale – Teacher edition (SCS-T), in two samples of children growing up with varied economic resources/conditions, cultural norms, and educational experiences, namely Pakistan (*N* = 396) and Sweden (*N* = 309).

**Methods:**

Participants were aged 4–6 years old. The study design was cross-sectional.

**Results and Discussion:**

Using structural equation modelling, bi-factor confirmatory factor analysis models implying shared variance, among all items and domain-specific shared variance, among the prosocial items, emotion regulation items, and academic skills items resulted in good fitting models in each respective sample. Invariance testing across samples revealed a subset of items from each factor structure with partial scalar invariance, whereby five items had equal thresholds and could be comparable across the two samples. Thus, results provided partial support for hypotheses 1, 2, and 3, in that the posited three factor model (H1) was not clearly supported and a bi-factor model evidenced the best fit, among tested models, for both samples. Further, partial scalar invariance (H3) was found for five items out of 25 items, concerning social competence and academic skills. In regards, to the posited research question, the results of Z-tests showed significant (*p* < 0.001) latent mean differences between the samples. Compared to the Swedish sample, the Pakistani sample was 1.80 units lower on social competence (*z* = −6.41, *p* < 0.001) and 1.86 units lower on academic skills (*z* = −7.87, *p* < 0.001). The implications of these findings in light of efforts to promote positive child development in diverse parts of the world are considered.

## 1. Introduction

The development of young children’s social emotional competence (SEC) is a complex, dynamic process that brings together children’s actions, emotions, thoughts, and takes place internally (within the child, intra-individually), as well as within an interpersonal context (Domitrovich et al., 2017; Rademacher and Koglin, 2019; Sauve and Schonert-Reichl, 2019). Core constructs involved in SEC include “… inhibition of impulsive behavioral responses, awareness and regulation of feelings, accurate perception of the perspectives of others, correct identification of problems, and development of positive and informed problem solutions and goals” (Riggs et al., 2006, p. 300). Children’s SEC is essential to gaining insight into and benefiting from social interactions with other children and adults in school and at home (Riggs et al., 2006). Indeed, the importance of SEC was highlighted in a progress report on the sustainable development goals (SDG 4) by 2030, in that aspects of social emotional competences, such as self-regulation and social skills, were singled out as having global relevance to today’s young people and viewed as vital to occupational success in many parts of the world (United Nations Educational, Scientific and Cultural Organization [UNESCO], 2016).

The importance of SEC is also supported by an expanding number of descriptive and experimental studies that connect SEC with better concurrent and prospective adjustment in children and adolescents (e.g., Taylor et al., 2017; Sauve and Schonert-Reichl, 2019; Yang et al., 2019). As a case in point, a meta-analysis of 82 school-based, universal social emotional learning (SEL) programs with at least a 6 month follow up found evidence in support of significantly improved social–emotional skills, attitudes, and well-being (Taylor et al., 2017). In a meta-analysis focused on young children (three to five-year olds) who lived in poverty, 29 studies were identified (randomized controlled and quasi-experimental trials of SEL interventions) and results showed that well implemented SEL interventions provided a significant boost to children’s SEC in comparison to children attending preschool without an explicit SEL instruction (Yang et al., 2019).

While these meta-analyses (Taylor et al., 2017; Yang et al., 2019) speak to the short-term benefits of SEL interventions, as well as the importance of SEC itself, a handful of long-term follow ups of early SEL interventions in preschool aged children illustrate the potential of these interventions to yield enduring benefits (e.g., Watts et al., 2018, 2020; Bierman et al., 2021). For example, the Head Start REDI (Research-based Developmentally Informed; Bierman et al., 2008a,b) combined an SEL curriculum with literacy training (*N* = 356) during preschool. In REDI, immediate intervention benefits were found for vocabulary, literacy skills, emotional knowledge, and improved social cognitions (Bierman et al., 2008b). Five years after the intervention, analyses showed longer-term intervention-related benefits on academic outcomes. Children who began behind their peers in executive functioning skills at the beginning of the REDI trial particularly benefited from the intervention across a range of outcomes (Sasser et al., 2017), and a long-term follow up into middle to late adolescence found intervention-related benefits on several key outcomes such as reduced prevalence and severity of conduct problems and emotional symptoms (Bierman et al., 2021).

With a promising empirical foundation for SEL in many parts of the world, efforts to foster the development of children’s SEC are taking place globally and with a growing focus on supporting SEL in schools as well as in communities (Hecht and Shin, 2015; Torrente et al., 2015). Such efforts can take the form of specific programs or initiatives (e.g., World Health Organization’s Life Skills framework or UNICEF’s Child Friendly Schools) or can be integrated into a nation’s educational policy and practice (Torrente et al., 2015). Diversity exists within and across nations in terms of how SEL is motivated and implemented in schools – for example, as a part of values or civic education or as an evidence-based means to achieve academic success and an improved transition to adulthood (Torrente et al., 2015).

Children’s social emotional development occurs in a changing historical, social, and cultural context (Hecht and Shin, 2015), whereby cultural norms and values with respect to social, emotional, and behavioural development may affect the expression and development of specific social behaviours (e.g., emotional expression and prosocial behaviour; Louie et al., 2015). It should also be noted that in terms of the diversity that we expected in children’s SEC, a guiding theory in this area was Bornstein’s (2017) specificity principle, which allows for an empirical testing of general principles about human development with an emphasis on examining the potentially unique as well as shared person-context interactions the produce development in various parts of the globe, across settings and historical time periods.

In order to realize the full benefits that well implemented SEL interventions can confer from a global standpoint, it is important to understand the diversity in the expression and functioning of children SEC throughout the global and particularly in countries in which the research literature on this topic is not extensive (Cefai et al., 2022). Further, at the heart of SEL interventions is the examination of intervention-related change, which is in part dependent on SEC measures that are theoretically grounded, psychometrically sound, useful, and culturally relevant (Cefai et al., 2022). Practically, this involves examining the psychometric properties of key instruments with diverse children. This also calls for open and free access to high quality instruments that combine sound psychometric properties with availability in a variety of languages, and culturally appropriate with good ecological validity. The SEL field has not yet realised its potential to have a global public health and educational impact; one of the missing pieces involves accessible, high-quality measurement of children’s SEC. Indeed, “several skills have been measured for decades, although mostly from a research angle, in high income countries and without cross-country comparability” (United Nations Educational, Scientific and Cultural Organization [UNESCO], 2016, p. 253).

### 1.1. The Social Competence Scale – Teacher edition

The Social Competence Scale – Teacher edition (SCS-T) was developed for use in the Fast Track study (Conduct Problems Prevention Research Group [CPPRG], 1995; Sorensen et al., 2016). The SCS-T partly consists of items from related scales (i.e., Kendall and Wilcox, 1979; Clark et al., 1985) and also includes newly created items that fit the Fast Track study’s wider aims and focus. The SCS-T can be completed by children’s teachers and has 25 items designed to measure prosocial/communication skills, emotional self-regulation, and academic skills.

The theory that guided the present study was CASEL’s five competence domain model, and the five domains consist of self-awareness, self-management, relationship skills, social awareness, and responsible decision making (Weissberg et al., 2015). The SCS-T is relevant to two domains in the CASEL model (Weissberg et al., 2015) – specifically the self-management domain (as indexed by emotional regulation skills items), which concerns intrapersonal skills; in other words: How do I get along with myself? (Domitrovich et al., 2017). “Competence in the self-management domain requires … the ability to delay gratification, manage stress, control impulses, and persevere through challenges…” (Weissberg et al., 2015, p. 6).

The SCS-T also concerns the relationship skills domain in the CASEL model, namely those interpersonal skills “… needed to successfully interact with others” (Domitrovich et al., 2017, p. 409). This is indexed in the SCS-T by items on the prosocial/communication skills scale. Based on the CASEL model (Weissberg et al., 2015), it would be expected that the self-management and relationship skills domains would be positively associated to one another but yet still be distinct facets of a multidimensional conception of social emotional competence, and that these two domains would be positively associated with important long-term outcomes for children and youth, such as being well prepared for school or doing well in school. A subscale also in the SCS-T concerns academic skills which is fitting with the research literature that shows a clear connection between the growth of varied social emotional competencies (e.g., self-management) and educational/academic achievement (e.g., Ursache et al., 2012; Hoffmann et al., 2020).

To illustrate the potential promise of the SCS-T, one can look to a study conducted with the Fast Track control group – this group included a normative group of children and children at risk for behavioural problems. The Fast Track control group did not take part in any Fast Track prevention activities (Jones et al., 2015). Fast Track was a multiyear intervention designed to reduce aggression and other problem behaviours over the long-term universally but also for a subgroup of children at elevated risk for behaviour problems (Jones et al., 2015). The Fast Track sample came from four areas of the United States (one rural and three urban) and children in the study were followed from a preschool age into young adulthood (Jones et al., 2015).

Results from an analysis of the Fast Track control group (*N* = 753, including high risk and normative children), showed that teacher-rated prosocial/communication skills (one subscale of the SCS-T) of the control group children, while the children attended kindergarten, were significantly related to several important outcomes at the age of 25 (the importance of prosocial/communication skills remained even when controlling for other factors such as socio-economic status, as well as neighbourhood, family and child background characteristics). Specifically, results showed that those children rated as having better skills in this domain in kindergarten by their teachers were more likely to graduate from high school and college, were more likely to have stable employment and to have fewer problems on young adult indicators of crime, substance use, and poor mental health (Jones et al., 2015). Although the SCS-T is a relatively brief teacher reported survey given at one point in time, early in a child’s development, it showed promising predictive validity in the Fast Track study. The study authors stated regarding the SCS-T:

“Our measure of social competence was a continuous composite from teacher observation that combined multiple social-behavioural scenarios for the child. This measure, although subject to measurement error, likely represents children’s social competence relatively well, because the teacher has been a daily observer in the classroom setting” (Jones et al., 2015, p. 2288).

The SCS-T has primarily been used in English speaking countries such the United States and Australia. In these studies, the SCS-T subscales (prosocial/communication skills, emotion regulation skills, and academic skills subscales) and a total score that combines all subscales typically evidence good internal consistency of Cronbach alpha coefficients of 0.80 or higher (U.S. studies: Conduct Problems Prevention Research Group [CPPRG], 1995; Corrigan, 2002; Australian studies: Duncombe et al., 2012; Havighurst et al., 2014). There is some evidence for the SCS-T’s predictive validity (Jones et al., 2015). As part of the Fast Track study, an item level exploratory factor analysis was conducted with kindergarten age ratings of children in the normative sample. Results indicated two factors, one factor combined many items from the prosocial/communication skills and emotion regulation skills subscales, and the other factor had academic skills items and some items from the other two scales. The two factors identified were significantly correlated at.83. In the SCS-T research literature, individual subscales are sometimes used (e.g., Duncombe et al., 2012; Jones et al., 2015) or all 25 items are combined into a single total score (e.g., Havighurst et al., 2014).

### 1.2. The present study

The present study aims to advance the measurement of children’s SEC and thereby play a supporting role to efforts to make SEL more accessible and widespread. This study tested the psychometric properties of a teacher-reported measure of children’s SEC, called the Social Competence Scale (SCS-T; Jones et al., 2015), in two samples of children who are growing up with varied economic resources/conditions, cultural norms, and educational experiences. This examination of the psychometric performance of the SCS-T (Jones et al., 2015) across the two study samples was based on a review of the research literature on culture and children’s SEL (Hecht and Shin, 2015), as well as theory – CASEL’s five competence domain model (Weissberg et al., 2015). A main aim of this study was to examine the psychometric properties of the SCS-T in contexts where it is likely to be used, in preschools within large urban centres located in two countries (Pakistan and Sweden). To the best of our knowledge, the SCS-T had not been used in Sweden or Pakistan before the present study was conducted.

### 1.3. Overview of Swedish and Pakistani contexts concerning child development

Sweden is a social democratic and largely secular nation and the present-day population is increasingly culturally and linguistically diverse (World Population Review, 2021). Sweden’s official languages include Swedish, Finnish, Yiddish, and Romani, whereby Swedish is the majority language. Sweden’s current population is approximately 10 million and children (between 0 and 14 years of age) account for 18% of this population. Sweden is wealthy by global standards (International Monetary Fund, 2021) and has social welfare values which emphasise an egalitarian society with high standards of well-being and quality of life (World Population Review, 2021). Sweden has a long-standing social welfare state whereby family policies are designed to support the dual earner family, gender equality, and high participation in the workforce; this is promoted, in part, through tax-funded social services (Ferrer-Wreder et al., 2020).

Pakistan is a Muslim majority country with a rich ethnic and cultural diversity. English is the official spoken language, but the national language is Urdu, which shares the official language status with English and is widely spoken and understood throughout the country. Nearly two-thirds of family systems in Pakistan are joint family structures (i.e., parents, children, their children and grandparents living together under one roof), however family systems are trending towards nuclear family structures in recent years with this trend moving faster in urban populations (Lodhi et al., 2021). Pakistan’s current approximate population is 226 million and, of this population, roughly 25% live under the poverty line (Humanium, 2021). There are 80 million children in Pakistan with fewer than 25% living in urban areas with access to fundamental needs (e.g., namely clean water, food, and health services). Pakistan provides free and compulsory education for children between the ages of five and sixteen, however, has the second highest number world-wide of children, aged five though 16, not attending school (44% of school-aged children; UNICEF, 2021). Currently 44% of the boys and 56% of the girls leave school before reaching the fifth grade (National Education Policy Review Team, 2007).

As noted by Khan (2018, p. 315) “the current curriculum approaches in Pakistan underscore language and cognitive skills and ignore social, emotional, cultural, physical and secular/spiritual development.” The educational system in Pakistan comprises four distinct school systems catering to different socio-economic strata of the society. These four systems are government public schools, for profit non-elite private schools, Cambridge school systems that are the elite private schools and denni madaris system that provide religious education. In public schools *katchi* classes remain common practice; whereby young unregistered children attend school with their older, registered sibling, and follow along with the existing lesson, within the same classroom in a multi-grade environment, and without any formal teaching directed to the younger unregistered child. The Pakistani government made a commitment (in 2002) to formalise *katchi* in public schools and increase the use of child-centred methods, due to a lack of resources and financing, children remain in crowded classrooms, with outdated syllabi, didactic and autocratic approaches to learning, and summative examinations (Rich-Orloff et al., 2007).

The private sector is the major provider of organized early childhood education in Pakistan. The for profit non-elite private sector schools have flourished enormously over the past few decades to fill the gap created by public sector education with reference to early childhood education. These schools serve middle-and lower-middle-class families and claim to offer quality preschool education. However, the quality of preschool education provided in these schools is also questionable.

### 1.4. Study hypotheses and research question

Consistent with theory (e.g., the CASEL model – Weissberg et al., 2015) and some of the prior research on the SCS-T from the U.S. (Conduct Problems Prevention Research Group [CPPRG], 1995; Corrigan, 2002), we hypothesised that the factor structure of the SCS-T–T subscales with these Swedish and Pakistani samples would have a two-factor structure for the prosocial/communication and emotional regulations skills and that academic skills would be a separate but positively related factor (i.e., a three factor structure overall).

*H1*: Across samples, the SCS-T factor structure would consist of three factors indexing aspects of prosocial/communication skills, emotional regulation skills, and academic skills.

*H2*: Across samples, associations between the three factors would be significant and positive.

*H3*: We expected that measurement invariance of the SCS-T factor structure would be found across the samples.

RQ. A research question that was posed (assuming measurement invariance was found) and was: Do mean levels of the measured social emotional competencies and academic skills significantly vary across samples?

This research question was posed given that the ways in which children may typically express these competencies and how they are perceived by their teachers may differ in form and function across the samples (Hecht and Shin, 2015) given the variation in participants’ preschool education, preschool teacher training, and variations in family structure and the wider social context.

## 2. Materials and methods

### 2.1. Participants

#### 2.1.1. Swedish sample

The child participants (*N* = 309) attended preschool in one of three municipalities in a large Swedish city. Participants were 53% male and 47% female. Participating children had an average age of four and half years old (*M = 4.56, SD = 0.55; N =* 309). Approximately 36% of the sample had a language, other than Swedish, as their first language and/or were multilingual (Swedish and another first language). First languages other than Swedish were varied and for example included: Arabic, Persian, Polish, Romanian, Russian, Somali, Spanish, Thai, and Turkish. Children’s family socio-economic status was not collected for this study. Preschools in three municipalities participated in this study and the median total income from employment and business income in 2016 (at the approximate time of data collection) for those living in two of these municipalities was below the county median and one other municipality was above the county median (Statistics Sweden, 2017).

#### 2.1.2. Pakistani sample

Child participants consisted of preschool children (*N =* 396) from for profit non-elite private sector preschools in middle-income neighbourhoods of the twin cities of Rawalpindi and Islamabad in Pakistan. Participating children were 49% male and 51% female. Participating children had an average age of four years old (*M* = 4.18, *SD* = 0.10).

Both, Sweden and Pakistan, were part of a limited number of countries who have participated in the PATHS program. The addition of the Pakistani sample was made possible by collaboration with a Pakistani research group led by Dr. Ayesha Inam.

### 2.2. Procedure

#### 2.2.1. Swedish study

The study inclusion criteria at the individual child level was that the children were four to six years old, and that the children wished to participate and their parents/guardians consented to have their children participate. Data reported on in this study are from two cross-sectional data collections. The wider aim of this program of research was to conduct a cultural adaptation of PATHS (Ferrer-Wreder et al., 2021) and test its effectiveness in a Swedish preschool context (Eninger et al., 2021). The SCS-T was part of a wider assessment battery and all data in this analysis were collected as part of the cultural adaptation process (Ferrer-Wreder et al., 2021) or before the intervention trial took place (at pre-test; Eninger et al., 2021).

The study procedure for the overall project was as follows: Three municipalities in a large Swedish city were approached to be in a wider intervention cultural adaptation and intervention trial study, and all municipalities accepted (Eninger et al., 2021). Municipalities largely contain urban and suburban neighbourhoods with the number of residents coming from non-Swedish backgrounds (i.e., born abroad or born in Sweden with both parents born abroad) ranging from approximately 18–58% of the respective municipalities’ populations. From these municipalities, 25 publicly or independently administered preschools were recruited to be in the study (open preschools, parent cooperative run preschools and family day homes were not recruited to be in the study). Roughly 10% of preschool children, in Sweden, attend independent preschools; independently administered preschools cannot charge fees greater than municipality run preschools (Skolverket, 2014). Within participating schools, individual children were recruited into the study. A Swedish regional ethics board approved this study (dnr. 2012/1714-31/5).

The present study analyses focus on teacher reports of the child participants’ prosocial and communication skills, emotional regulation skills, and academic skills (i.e., ratings on the SCS-T). As part of the wider study, research assistants also observed the children at play and gave child participants several tasks measuring, for example, social problem solving, theory of mind, emotional recognition, and aspects of executive functioning (Eninger et al., 2021). Following the completion of the child tasks and play observation, teachers were sent, by post, a survey and asked about the competencies and problem behaviours of the individual children participating in the project. Schools received one survey per participating child which was to be completed by the child’s teacher. Once the teachers had completed the surveys, schools returned the surveys in pre-stamped envelopes to the research team. All surveys returned by teachers for participating children were used in this analysis and any missing data were estimated (see data analysis plan for details).

Translation of the SCS-T, into Swedish, was guided by a committee approach (van de Vijver and Leung, 1997). Initial translation was done by a licensed psychologist familiar with the scales and assessment, and fluent in Swedish and English. Translated items were reviewed by another researcher with a PhD in psychology, and fluent in Swedish and English, to ensure the meaning of the items were preserved in the final translation.

Pakistani study. The study procedure and circumstances of the Pakistani study has several parallels to the Swedish study, in that recruitment in both studies took place first at the preschool level. However, in this case, instead of convenience sampling (as was used in the Swedish study), a purposive sampling technique was used in the Pakistani study to recruit seven private sector preschools (Inam et al., 2015; Inam, 2016). Individual children were then recruited into the study from participating schools. Both studies took place in large cities in the respective nations, and in this case all data are from the pre-test (i.e., cross-sectional data) of the intervention trial of a culturally adapted edition of the preschool PATHS program (Inam et al., 2015). As in the Swedish study, the SCS-T was part of a wider assessment battery completed by teachers for individual children participating in the PATHS trial (Inam et al., 2015). As part of the pretest, a total of 450 questionnaires were distributed to teachers and 54 questionnaires were discarded due to missing data. Only completed questionnaires were retained for analysis in this study. The Advanced Study and Research Board (AS & RB) of Quaid-i-Azam University Islamabad Pakistan approved this study.

As Urdu is the national language of Pakistan, and while sharing official status with English, it is the preferred and dominant language used for inter-communication between different ethnic groups. Din (2015) found that in Pakistan, learners at graduate and post-graduate level did not possess the required language proficiency as realized through poor language in their language proficiency tests, paper writing and general understanding of English text. Considering this, the translation of this scale was done in the national language. The translation of SCS-T into Urdu language was done through Brislin (1980)’s committee approach method using five steps, as follows:

Step 1: Evaluation of relevance of scale items for Pakistani children. The first step, before going to the translation phase, was to evaluate the items of the scales. Seven experts, comprising four preschool teachers and three academics, each with more than 20 years’ experience of working with young children in school settings, were contacted. Together, they reviewed and evaluated the relevance of the items to preschool children in this study. The items were found relevant to the behaviours of children usually observed in Pakistani schools.

Step 2: Forward translation of items. Next, a group of six bilingual experts were approached. Of these, two were school teachers having Master degrees in English and Education and a command of Urdu and English language; three were PhD scholars working in the field of developmental psychopathology and social psychology; and one was an educationist who had experience working with young children and also proficient in both languages. They translated the items keeping in view the conceptual equivalence. These translations were further reviewed to exclude any repetitions.

Step 3: Committee approach. A three-member committee reviewed and evaluated the most appropriate translation of the scale. The members were researchers and faculty members in the discipline of Psychology and having adequate experience in item construction and translation.

Step 4: Back translation of items. In this step, three bilingual experts were asked to back translate the Urdu version of the scale selected through committee approach. The minimum education of these bilinguals were Masters and they were not familiar with the original version of the scale.

Step 5: Final item selection. A committee of three members evaluated the three back translations and compared the original version and back translation. The items, which conveyed the closest meanings to the original scale and were deemed most appropriate, were retained. Twenty items were retained. The committee recommended the final version used in this study analysis for further use in research.

### 2.3. Teachers as reporters on children’s SEC in this study

Teachers in this study, in general were not considered to be study participants, and thus no demographic information was recorded for teachers as part of the child assessment. However, it can be noted that in Swedish preschools, it is typical to have teachers and other helping staff who vary in their education in education and child development with some teachers regularly having undergraduate to masters level university degrees and with other helping staff having a three year upper secondary school training in the area to those with little formal education (Panagiota et al., 2017). In Pakistan, generally in for profit non-elite private sector schools, teachers have academic qualification from undergraduate to graduate levels in different disciplines but not specifically early childhood education (Khan, 2018).

### 2.4. Measures

Social Competence Scale – Teacher Version contains 25 items that are designed to index three subscales, namely: Prosocial/Communication Skills (Items 9, 13, 19, 20, 22, 23, 24, and 25), Emotional Regulation Skills (Items 2, 3, 6, 7, 8, 11, 12, 14, 16, and 18), and Academic Skills (Items 1, 4, 5, 10, 15, 17, and 21; Conduct Problems Prevention Research Group [CPPRG], 1995; Corrigan, 2002). In addition to the subscale scores, a score for the combined Prosocial/Communication items and Emotional Regulation items can be calculated, and a total score on all 25 items can also be reported (Conduct Problems Prevention Research Group [CPPRG], 1995; Corrigan, 2002). Individual subscale scores, the combined score, and the total score are calculated as the mean of item responses (Conduct Problems Prevention Research Group [CPPRG], 1995; Corrigan, 2002). In the instructions for this measure it is noted that if more than half of the items for a scale are missing responses, the total or sub scale score should not be calculated (Conduct Problems Prevention Research Group [CPPRG], 1995; Corrigan, 2002). Measures with all responses missing are not scored (Conduct Problems Prevention Research Group [CPPRG], 1995; Corrigan, 2002). As noted, 20 items were used in this study: Prosocial/Communication Skills items 13, 19, 20, 22, and 25; Emotional Regulation Skills items 2, 3, 6, 8, 11, 12, 14, 16, and 18; and Academic Skills items 4, 5, 10, 15, 17, and 21. All completed items were retained for analysis in the Swedish study (missing data were estimated) and only completed surveys were retained in the Pakistani study.

The SCS-T at the item level describes a behaviour that a child may display at school and examples include: “Works well in a group” and “Can give suggestions and opinions without being bossy.” The teacher assesses how well each statement describes the child. Responses are coded on a five-point Likert scale: “Not at all (0),” “A little (1),” “Moderately well (2),” “Well (3),” and “Very well (4)” (Conduct Problems Prevention Research Group [CPPRG], 1995; Corrigan, 2002).

As noted in the introduction, the SCS-T in U.S. and Australian studies has shown good scale reliability (internal consistency.80 or higher; e.g., Conduct Problems Prevention Research Group [CPPRG], 1995; Corrigan, 2002). The construct validity of the SCS-T with U.S. children as part of the Fast Track study showed a two-factor structure (using exploratory factor analysis at the item level), one combined prosocial/communication skills and emotion regulation skills factor and one factor with academic skills, with the two factors showing a high positive correlation (i.e., *r* = 0.83). The psychometrics for the SCS-T with these study samples are described in the results section.

### 2.5. Statistical analysis

All analyses were performed using SPSS 23.0 and Mplus 8.4 (Muthén and Muthén, 1998–2017). We established and explored (in each sample separately) the construct validity (i.e., factor structure) and internal consistency in stages: to examine H1 and H2 we (1) evaluated a three-factor model of SCS-T items; (2) compared a one-factor global model, and (3) a bi-factor model that includes the three specific factors and the global factor, and lastly (4) assessed the internal consistency of SCS-T scales. Once we had established good construct validity in each sample, in order to examine H3, we tested for measurement invariance of the SCS factor structure across samples. If invariance was established then it was possible to test the research question (RQ), SCS-T scores across the samples would be examined to determine if there were significant mean level differences in social emotional competence or academic skills.

#### 2.5.1. Confirmatory factor analysis

**Figure 1:**
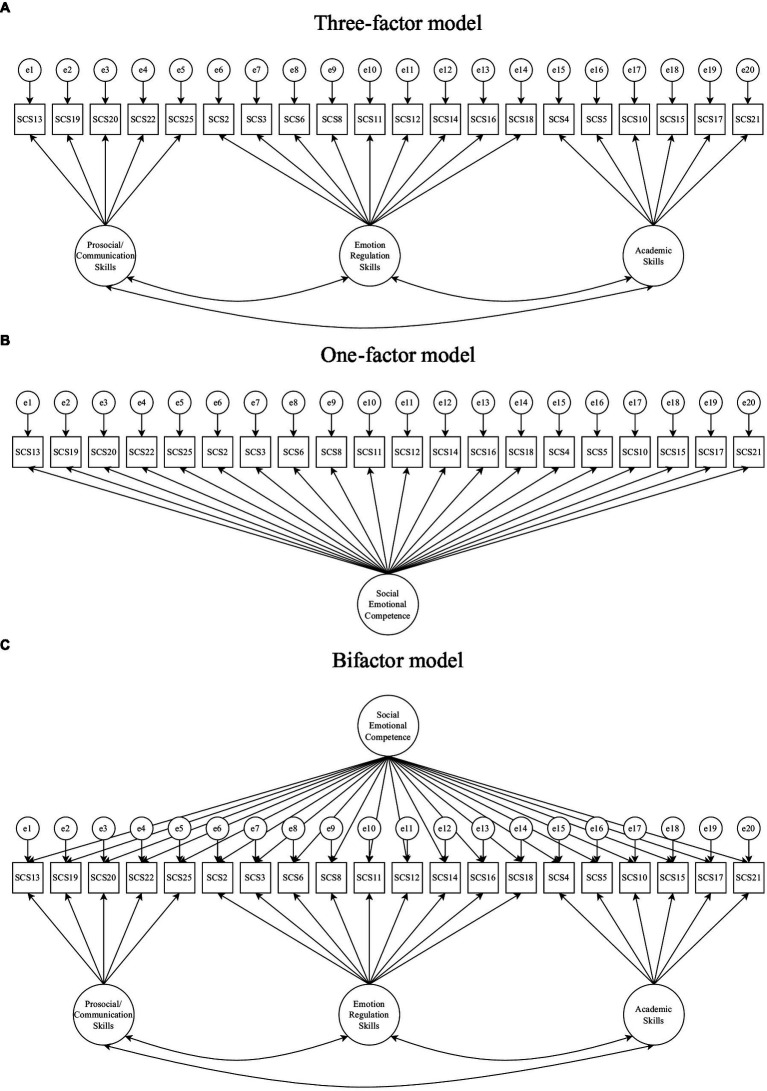
These are the three **(A, B, C)** factor models used for evaluating the factor structure of the SCS-T.

Consistent with theory and prior psychometric studies of the SCS-T, three measurement models (a three-factor, global one-factor and bifactor model) for the SCS-T were evaluated using structural equation modelling (SEM) based CFAs. These models are presented in Figure 1. For all models, we used a robust means-and-variance-adjusted weighted least squares (WLSMV) estimator to accommodate the Likert-scale item data, and we scaled the latent factors using the fixed factor method in which the variance of each latent factor is fixed at 1.00 (Little, 2013).

As shown in Figure 1, the three-factor model included the latent factors of: prosocial/communication skills, emotion regulation, and academic skills. The global one-factor model collapsed prosocial, emotion regulation skills, and academic skills into one global factor of social emotional competence. The bifactor model specified that there is shared variance among all items and domain-specific shared variance among the prosocial/communication skills items, among the emotion regulation skills items, and among the academic skills items. Model fit indices included the chi-square goodness of fit test, the root mean square error of approximation (RMSEA), the comparative fit index (CFI), and the standardised root mean square residual (SRMR). Good fit was defined by a chi square value of *p* ≥ 0.05, RMSEA < 0.05, CFI ≥ 0.95, TLI ≥ 0.95, and SRMR < 0.05. Chi square difference tests were used to compare nested models.

#### 2.5.2. Measurement invariance

Multigroup analyses conducted in Mplus, starting with a configural invariance model. Configural invariance is when the same factor structure holds across groups, in this case samples. A metric invariance model was then specified. Metric invariance includes both equivalent factor structures and equal factor loadings. Finally, based on the results from the first two steps, a partial scalar invariance model was specified. Scalar invariance includes equivalent factor structures, equal factor loadings, and equal intercepts or thresholds.

#### 2.5.3. Latent mean differences between samples (*z*-tests)

Finally, we compared the Swedish and Pakistani samples on the means of the latent variables that exhibited partial scalar invariance: social competence and academic skills.

## 3. Results

### 3.1. Confirmatory factor analysis results

This analysis addressed H1 which posited a three-construct factor structure consisting of prosocial/communication skills, emotion regulation skills, and academic skills. Table 1 presents the model testing and model comparison results. In the Swedish sample, the 1-factor model did not meet any criteria for good fit, whereas both the 3-factor and bifactor models had acceptable CFI, TLI, and SRMR values but not RMSEA values. Chi square difference testing indicated that the bifactor model fit better than the 3-factor, which fit better than the 1-factor model. In the Pakistani sample, all three models had acceptable CFI, TLI, and SRMR values but not RMSEA values. Similar to the Swedish sample, chi square difference testing indicated that the bifactor model fit better than the 3-factor, which fit better than the 1-factor model. Based on these results (particularly the chi square difference tests), H1 was not supported and a bifactor factor structure appears to be the best fitting factor structure of the tested models in both samples. The bi-factor model implies that there is shared variance among all items (i.e., social emotional competence) *and* domain-specific shared variance among the prosocial/communication skills items, among the emotion regulation skills items, and among the academic skills items. Table 2 shows the factor correlations in each sample which ranged from 0.26 to 0.41 in the Swedish sample and 0.40 to 0.65 in the Pakistani sample. Thus, hypothesis 2 was supported, in that associations between the three factors (prosocial/communication skills, emotion regulation skills, and academic skills) were significant and positive.

**Table 1:** Model fit and model comparison.

Model	Model fit								Model comparison			
	*χ* ^2^	df	*p*	CFI	TLI	RMSEA	CFit	SRMR	vs.	*χ* ^2^_diff_	df	*p*
*Sweden (n = 307)*												
1. 1-factor	1476.983	170	<0.001	0.937	0.930	0.158 [0.151, 0.166]	<0.001	0.069	2	194.947	3	<0.001
2. 3-factor	948.244	167	<0.001	0.962	0.957	0.123 [0.116, 0.131]	<0.001	0.049	3	303.583	20	<0.001
3. Bifactor	546.051	147	<0.001	0.981	0.975	0.094 [0.086, 0.103]	<0.001	0.035	–	–	–	–
*Pakistan (n = 396)*												
4. 1-factor	659.764	170	<0.001	0.973	0.970	0.085 [0.078, 0.092]	<0.001	0.040	5	90.049	3	<0.001
5. 3-factor	540.713	167	<0.001	0.979	0.977	0.075 [0.068, 0.082]	<0.001	0.036	6	132.088	20	<0.001
6. Bifactor	418.373	147	<0.001	0.985	0.981	0.068 [0.061, 0.076]	<0.001	0.030	–	–	–	–
*Multigroup (n = 703)*												
7. Conf.	958.273	294	<0.001	0.983	0.978	0.080 [0.075, 0.086]	<0.001	0.032	–	–	–	–
8. Metric	985.435	329	<0.001	0.983	0.981	0.075 [0.070, 0.081]	<0.001	0.034	7	78.789	35	<0.001
9. Metric-P	968.541	309	<0.001	0.983	0.979	0.078 [0.072, 0.083]	<0.001	0.033	7	14.449	15	0.492
10. Scalar-P1	1228.027	363	<0.001	0.978	0.977	0.082 [0.077, 0.087]	<0.001	0.038	9	304.188	54	<0.001
11. Scalar-P2	971.343	324	<0.001	0.983	0.980	0.075 [0.070, 0.081]	<0.001	0.033	9	19.360	15	0.199

CFI, Comparative Fit Index; TLI, Tucker-Lewis Index; RMSEA, Root Mean Square Error of Approximation; CFit, *p*-value for test of closeness of fit; SRMR, Standardized Root Mean Square Residual.

**Table 2:** Estimated factor loadings and factor correlations.

Item	Swedish				Pakistani			
	PCS	ERS	AS	SEC	PCS	ERS	AS	SEC
*Factor loadings*								
13.	0.19 [0.12, 0.27]			0.79 [0.74, 0.84]	0.16 [0.09, 0.23]			0.73 [0.68, 0.78]
19.	0.48 [0.40, 0.55]			0.76 [0.71, 0.82]	0.35 [0.27, 0.43]			0.69 [0.63, 0.75]
20.	0.51 [0.43, 0.59]			0.81 [0.76, 0.86]	0.59 [0.50, 0.67]			0.66 [0.60, 0.73]
22.	0.48 [0.41, 0.55]			0.79, [0.73, 0.84]	0.45 [0.36, 0.54]			0.72 [0.66, 0.77]
25.	0.35 [0.27, 0.42]			0.79 [0.74, 0.84]	0.28 [0.21, 0.34]			0.73 [0.68, 0.79]
2.		0.68 [0.59, 0.78]		0.63 [0.54, 0.72]		0.53 [0.44, 0.63]		0.62 [0.55, 0.69]
3.		0.71 [0.63, 0.80]		0.58 [0.49, 0.68]		0.55 [0.46, 0.64]		0.58 [0.52, 0.65]
6.		0.31 [0.21, 0.41]		0.85 [0.80, 0.90]		0.34 [0.25, 0.43]		0.77 [0.72, 0.81]
8.		0.16 [0.06, 0.26]		0.80 [0.76, 0.85]		0.09 [0.03, 0.16]		0.72 [0.67, 0.77]
11.		0.44 [0.34, 0.53]		0.75 [0.68, 0.81]		0.18 [0.12, 0.25]		0.74 [0.70, 0.79]
12.		0.14 [0.04, 0.25]		0.82 [0.78, 0.87]		0.06 [0.01, 0.11]		0.79 [0.75, 0.84]
14.		0.06 [−0.03, 0.15]		0.87 [0.83, 0.91]		0.03 [−0.02, 0.07]		0.71 [0.66, 0.76]
16.		0.14 [0.04, 0.25]		0.89 [0.86, 0.93]		0.06 [0.01, 0.10]		0.75 [0.70, 0.79]
18.		0.42 [0.32, 0.51]		0.78 [0.72, 0.84]		0.24 [0.17, 0.32]		0.75 [0.70, 0.80]
4.			0.69 [0.62, 0.77]	0.58 [0.50, 0.67]			0.53 [0.44, 0.62]	0.69 [0.63, 0.75]
5.			0.56 [0.49, 0.63]	0.71 [0.64, 0.78]			0.41 [0.32, 0.51]	0.69 [0.64, 0.75]
10.			0.52 [0.46, 0.59]	0.75 [0.70, 0.81]			0.37 [0.28, 0.46]	0.76 [0.70, 0.81]
15.			0.41 [0.35, 0.48]	0.84 [0.79, 0.88]			0.25 [0.17, 0.33]	0.74 [0.69, 0.79]
17.			0.45 [0.38, 0.51]	0.74 [0.68, 0.80]			0.28 [0.19, 0.36]	0.80 [0.76, 0.84]
21.			0.38 [0.30, 0.45]	0.83 [0.79, 0.88]			0.19 [0.12, 0.25]	0.76 [0.71, 0.81]
*Factor correlations*								
PCS	–	0.41 [0.24, 0.59]	0.39 [0.24, 0.53]	0.00 [0.00, 0.00]	–	0.53 [0.40, 0.66]	0.40 [0.24, 0.56]	0.00 [0.00, 0.00]
ERS		–	0.26 [0.08, 0.45]	0.00 [0.00, 0.00]		–	0.65 [0.53, 0.78]	0.00 [0.00, 0.00]
AS			–	0.00 [0.00, 0.00]			–	0.00 [0.00, 0.00]
SEC				–				–

All estimates are standardized. 95% confidence intervals are in brackets. PCS, prosocial/communication skills; ERS, emotion regulation skills; AS, academic skills; SEC, social emotional competence.

### 3.2. Invariance results

These analyses addressed H3. As shown in the Multigroup section in Table 1, the metric invariance model (Model 8) fit significantly worse than the configural invariance model (Model 7). As a result, modification indices were used to identify factor loadings that were not equivalent. All factor loadings for the general factor and the loading for one of the emotion regulation items (SCS6) were freed to vary across groups. The difference in fit between this partial metric invariance model (Model 9) and the configural invariance model was not statistically significant. Next, a partial scalar invariance model was then specified. Scalar invariance includes equivalent factor structures, equal factor loadings, and equal indicator intercepts (or, in this case, thresholds) whereas, partial scalar invariance means that some items do not have scalar invariance. The initial partial scalar invariance model added equal thresholds across groups for all items except SCS6. This model (Model 10) fit worse than the partial metric invariance model. Modification indices were used to identify thresholds that were not equivalent and were then freed. The final result was a partial scalar invariance model (Model 11) in which five items had equal thresholds and could be compared across the two samples. The five items were SCS19, SCS22, and SCS25 from the 5-item prosocial/communication skills subscale and SCS15 and SCS21 from the 6-item academic skills subscale. Based on these results, H3 (full measurement invariance) was not supported. Instead, results indicated that items measuring prosocial/communication skills and academic skills were partially invariant, but that items measuring emotion regulation skills and global social emotional competence were noninvariant. Consequently, mean levels of emotion regulation skills and global social emotional competence cannot be compared across Swedish and Pakistani groups.

Table 2 presents the estimated standardized factor loadings and factor correlations in both the Swedish and Pakistani groups. Across both groups, factor loadings were generally stronger for the global social emotional competence factor than the domain specific factors, ranging from 0.58 to 0.89 for the Swedish group and from 0.58 to 0.80 in the Pakistani group. Many items only weakly loaded onto domain specific factors, including several with standardized factor loadings of less than.20.

### 3.3. Latent mean differences between samples (*z*-tests)

#### 3.3.1. This analysis addressed the research question

Compared to the Swedish group, the Pakistani group was 1.80 units lower on prosocial/communication skills (*z* = −6.41, *p* < 0.001) and 1.86 units lower on academic skills (*z* = −7.87, *p* < 0.001). There were indeed some significant across sample differences on the five items.

## 4. Discussion

The findings in this study provide initial support for reliability, factor structure (e.g., construct validity) and feasibility of the SCS-T for use with children in Swedish and Pakistani urban preschools. The implications of our findings, in light of efforts to promote positive child development in diverse parts of the world, lend support for the importance of establishing psychometric performance of SEL instruments across cultural and societal contexts where children are living.

Our initial hypotheses (H1 and H2) were partially supported by the fit indices, factor loadings, and latent construct correlations, as well as composite reliabilities, based on the final SCS-T bi-factor model in, both samples; even though the children in these samples are growing up with varied economic resources/conditions, cultural norms, and educational experiences. Although a three factor model was not clearly supported by the results (H1 not supported in full) a bi-factor model had the best fit, across the tested models, across samples. A bi-factor model implies that there is shared variance among all items as well as domain-specific shared variance among the prosocial items, emotion regulation items, and the academic skills items; or in other words, that all items load onto a general/global factor, as well as a series of skill-specific factors. These findings are in line with the CASEL five competence domain model’s (Weissberg et al., 2015) associations between social emotional competencies, and further highlights just how interrelated these constructs/aspects of development are. Past research on the SCS-T has shown some diversity in the factor structures found for this instrument (e.g., Conduct Problems Prevention Research Group [CPPRG], 1995; Gouley et al., 2008) and some change in factor structure (towards a more distinction between social emotional scales by teacher raters) in older children (those in early elementary school; Gouley et al., 2008), and thus more research on this promising instrument is clearly needed across early childhood.

Additionally, we were able to achieve *partial* scalar invariance (H3); whereby five items from the SCS scales’ social competence and academic subscales allowed for comparison between samples. When compared (RQ), we found that the Pakistani sample had lower social competence and academic skills than the Swedish sample (on a subset of comparable SCS-T items). Child development is tied to the specific cultural and social contexts in which the child is developing (e.g., ECEC/daycare). The cultural and social context provides specific socialisation contexts that interact with the unique abilities and competencies of the developing child (Albert and Trommsdorf, 2014). Children living in cultures with predominantly joint family structures (as was the case, traditionally, in Pakistan), tend to exhibit more prosocial behaviours, than children who live in cultures with predominantly nuclear family structures (Singh et al., 2014). Since Pakistan’s urban population has trended largely towards the nuclear family system (like that in Sweden; Lodhi et al., 2021), parents are more reliant on the state or private care for early childhood/day-care. Unfortunately, Pakistan’s ECECs are underfunded and suffer a shortage of educated teachers, limiting access to high-quality ECEC (Khan, 2018); whereas Sweden has near-universal access to high-quality ECEC (Swedish National Agency for Education, 2016). Although the Pakistani government made a commitment (in 2002) to increase the use of child-centred methods in ECEC and formalise curriculum for ECEC, due to a lack of resources and financing, this has not yet been fully possible (Rich-Orloff et al., 2007; Khan, 2018).

Theoretically rooted and empirically sound assessments are best practice in educational settings to identify children’s behavioural and emotional strengths and difficulties (Greenberg and Rhoades, 2010). Measurement can help to inform researchers, educators, and practitioners; and promote children’s social emotional development in a global context with consideration of unique social and cultural settings (Tam and Lee, 2010). The implications of our findings highlight (1) the importance of establishing psychometric performance (i.e., validity and reliability) of SEL instruments across diverse children living in differing contexts and (2) the promise of using SEL measurement in culturally and linguistically diverse populations.

SEL assessments typically are informed by children themselves, their parents, teachers/school staff and peers (Wigelsworth et al., 2010). Teacher rating scales are increasingly being used to assess social–emotional competencies using the CASEL model as a viable other-rater assessment in school settings especially for student traits and performance (Poropat, 2014), and are particularly appropriate when measuring observable behaviour (Merrell, 2009). Teachers benefit from using their collective experiences with same-aged peers as a reference point for assessments of students which may contribute to teacher rating scales having high discriminative validity (Humphrey et al., 2008).

As is the case with all measurement, biases exist in teacher report measures including on the individual level of students (i.e., reporting more favourably for students they like) and/or at the classroom level as a result of teacher variance (i.e., leniency or severity bias whereby individual teachers rate students more positively or negatively than another teacher might) (Merrell, 2009). Notwithstanding, as the field of monitoring and evaluation of SEL continues to develop globally, teachers represent an important source for measurement, optimally when triangulating results with additional data from parents, children and/or peers (Wigelsworth et al., 2010).

### 4.1. Study limitations and strengths

This study is cross-sectional and therefore any causal relations between any of the study constructs, or developmental changes across time cannot be inferred. Further, both samples consisted of children coming from schools who wished to participate and were interested in receiving access to, and training in, a social emotional learning curriculum (e.g., Swedish sample) or receiving additional classroom management training (e.g., Pakistani sample). Even though there were some important similarities in the sample study procedures (i.e., same instrument was used in the two samples and the child study participants were of a similar age, gender distribution, living in an urban context, and attending preschool), there were also variations in some regards (e.g., translation process of the instrument, school recruitment procedure, handling of missing data). Further, aspects of culture or features of the educational context at participating preschools were not directly measured in this study, and within each sample there is linguistic and cultural diversity. The samples in this study were not intended to be nationally representative. Therefore, any cross-sample findings are generalizable to the present samples, and any further generalisation of findings to Swedish or Pakistani children in general should not be made in the absence of research that specifically has the intention to examine this research topic in nationally representative samples of children.

Despite these limitations, clear study strengths include the first time use and examination of the psychometric properties of the SCS with children attending preschools in Sweden and Pakistan. Thus, opening up the possibility of the use of the SCS more widely in Swedish and Pakistani early childhood educational contexts. Other important advances are the free availability of this psychometrically useful instrument in two additional languages (Swedish and Urdu), see data availability statement for details about how to access these editions of the instrument, as well as information about how to possibly score this instrument with children attending preschool in the respective countries. Further studies are needed to have greater confidence in the factor structure of this instrument in the wider population, yet the present study provides a starting point for this future line of inquiry.

This study also highlights the need for more consideration into the situation in which results point to partial invariance and guidelines for interpretation of such findings. Regarding the invariance testing conducted with the SCS-T in this study, standards for acceptability of partial invariance vary from notions that half of all items (Steenkamp and Baumgartner, 1998) to a majority of items (Vandenberg and Lance, 2000) on a factor should be invariant. However, these guidelines are not supported by empirical evidence and there remains ambiguity in the field regarding best practice for testing and interpreting partial invariance (Putnick and Bornstein, 2016). Future research evaluating the usefulness of partially invariant scales and the development of empirically based standards and thresholds for partially invariant scales will be valuable next steps in advancing the global study of social competence scales. Because study results pointed to relatively weak evidence of invariance in that five items were invariant across contexts (out of a total twenty-five), it is important to conduct future research in these study contexts, and this future research should employ mixed-method approaches, such as having teachers discuss how they interpret the meaning of items and their relevance to their views of the constructs measured by the SCS-T.

At a fundamental level, this study also helps to develop increased insight into variations in children’s SEC. We entered this study with an expectation consistent with Bornstein’s (2017) specificity principle. Namely, we expected that there could be evidence for both general commonalities as well as specific features of SEC in our samples that were unique and non-comparable. For example, consistent with the idea of generalities, the study results indicated that the constructs of SEC examined in study could be reliably and validly measured (i.e., with a bi-factor model, a general cross sample finding) and that associations among the respective facets of SEC were positively and significantly related to one, which is consistent with for example the CASEL SEC theory. But equally inline with the idea of uniqueness, other study results indicated that wide scale, cross sample comparisons were not tenable in light of the invariance testing results (for all but for five out of 25 items). Thus, we were open to the results to guide us in finding that in general these constructs are of importance across samples and can be measured in a similar way, but direct comparisons across the samples was not warranted likely due to important variations in the life conditions of children, which we have little insight into given the lack of measurement of the preschool context and wider context of these children’s daily lives and life conditions. Bornstein’s (2017) specificity principle challenged us as researchers to consider children’s SEC could express itself in a unique and similar ways among the study samples and that systematic investigation into the measurement of children’s SEC across different cultural and societal contexts must be carefully considered and culturally valid measurement is to be strived for and considered critically. Many of the instruments developed for use in the field of SEL have been first developed in western, educated, industrialised, rich, and democratic (WEIRD) countries (Henrich et al., 2010), and collaborative studies such as the present one with research teams conducting studies in varied contexts are important to widening the field of SEC so that is it is increasingly able to speak to the diversity of child development.

## 5. Conclusion

Social competence is increasingly recognized as a dependable predictor of an array of public health outcomes including education, employment, criminal activity, substance use, and mental health and well-being across the life-course (e.g., Jones et al., 2015). The availability of valid and reliable measurements of social competencies within and between diverse cultural contexts is core to informed decision making surrounding prioritisation and implementation of social emotional learning interventions. This study provides evidence supporting the Swedish and Urdu translations of the SCS-T, allowing for their use and further psychometric development and testing in the respective contexts. Additionally, the results of the study related to the invariant and non-invariant items isolated through the multi-group comparison can inform and facilitate additional theoretical, descriptive, and comparative studies exploring cultural similarities and differences of the construct of social competence. Continued testing and exploration of social competencies in a global context is warranted.

## Data availability statement

Requests for de-identified data for the study analyses in this article can be made to the corresponding author by qualified researchers (PhD.) with Swedish and Pakistani ethical approval for secondary data analysis. This procedure complies with the ethical approval for the studies in this article.

## Ethics statement

The Swedish study had ethical approval from a Regional Ethics Review Board in Sweden (dnr. 2012/1714-31/5). The Pakistani Study had ethical approval from the Advanced Study and Research Board (AS & RB) of Quaid-i-Azam University, Islamabad Pakistan. Written informed consent to participate in this study was provided by the participants’ legal guardian/next of kin.

## Author contributions

ST, AK, KE, AI, LF-W, and LE participated in the manuscript conceptualization and writing. LF-W, ST, AI, and KE: data curation. ST and KE: formal analysis. LE, LF-W, and AI: funding acquisition. ST, AI, LF-W, and LE: investigation. LE, LF-W, and AI: methodological design. ST, LE, LF-W, and AI: project administration. All authors: writing—review and editing, and approve this publication and are accountable for the accuracy of the work in this article. All authors contributed to the article and approved the submitted version.

## Funding

Funders of the Swedish Study included: combined funding from the Swedish Council for Working Life and Social Research, the Swedish Research Council, Formas, and VINNOVA (dnr: 259-2012-71), the Clas Groschinsky Memorial Fund, and Stockholm University’s Centrum för Kompetensutveckling inom Vård och Omsorg (CKVO).

## Conflict of interest

The authors declare that the research was conducted in the absence of any commercial or financial relationships that could be construed as a potential conflict of interest.

## Publisher’s note

All claims expressed in this article are solely those of the authors and do not necessarily represent those of their affiliated organizations, or those of the publisher, the editors and the reviewers. Any product that may be evaluated in this article, or claim that may be made by its manufacturer, is not guaranteed or endorsed by the publisher.
